# Cherokee County Medical Society

**Published:** 1888-05

**Authors:** 


CHEROKEE COUNTY MEDICAL SOCIETY.—We are indebted to Dr. J. F. Johnson, Secretary, for an account of the organization of the Cherokee County Medical Society, and the proceedings of the first meeting. The physicians of Rusk and Alto met May 3, and organized by drafting a Constitution and By-Laws, and by electing Dr. A. H. McCord President, and Dr. J. F. Johnson Secretary. Dr. McCord read a paper which, on motion, was sent to DANIEL'S TEXAS MEDICAL JOURNAL for publication (the paper will appear in next issue).

Drs. Barton and Evans, of Alto, and Jameson and Spring, of Rusk, were appointed a Committee on Correspondence. Subjects selected: "Unusual Manifestations of Malaria," "Eczema,"

"Vomiting of Pregnancy," and "Indications for the Use of the Forceps." Adjourned to meet at Alto, 1st Tuesday in June.

Thus the good work goes on. We join Dr. Johnson in his appeal to the physicians of Cherokee to lend a helping hand, and to rally around the nucleus already organized.

---

WE tender our sincere acknowledgements to our esteemed confrere, Sim, of the Memphis *Medical Monthly*, for Cincinnati papers containing an account of the meeting of the A. M. A.

---

ERYTHROPLEIN, THE NEW LOCAL ANASTHETIC.—Dr. David Livingston, in his work on Africa, mentions a drug used by the natives, called *haya* or *hayab*. This is supposed to be the same "red drug," about which Oertel wrote from the west coast of Africa, the early part of this century. It was used by the negroes as an arrow poison, and to give to those accused of witchcraft. If the supposed criminal vomited within a few minutes after taking the drug, he was set free. Otherwise he was stoned and clubbed to death.

Professor L. Lewin, (*Wiener Medizinische Blaetter—St. Louis Medical and Surgical Reporter*), has recently investigated the properties of this drug, and announces that it is a local anæsthetic of great intensity, and destined to throw cocaine entirely in the shade.

A two per cent. aqueous solution of erythroplein, which is the active principle of *erythropleum judiciale*, not only completely anæsthetizes the mucous membrane, wherever it is applied, but its effects go deeper into the tissues, and lasts from six to ten hours. Injected under the skin of guinea-pigs, the tissues down to the deep muscles could be cut and lacerated to any extent without causing the least flinching or other evidence of pain. Death from an overdose is accompanied by convulsions.

A sample of the drug has been sent for by O. Samostz, druggist, of Austin.

For further information concerning this wonder in anæsthesia is awaited with impatience.

